# Effects of Phytoestrogens in Alleviating the Menopausal Symptoms: A Systematic Review and Meta-Analysis

**Published:** 2017

**Authors:** Nafiseh Saghafi, Masoumeh Ghazanfarpour, Ramin Sadeghi, Azadeh Hosseini Najarkolaei, Maryam Ghaffarian Omid, Afrooz Azad, Mahmood Bakhtiyari, Elnaz Hosseini Najarkolaei

**Affiliations:** a *Department of Obstetrics and Gynecology, Mashhad University of Medical Sciences, Mashhad, Iran. *; b *Student Research Committee, School of Nursing and Midwifery, Mashhad University of Medical Sciences, Mashhad, Iran. *; c *Nuclear Medicine Research Center, Mashhad University of Medical Sciences, Mashhad, Iran. *; d *Mahdiyeh Hospital, Shahid Beheshti University of Medical Sciences, Tehran, Iran*; e *Ghaem Hospital, Mashhad University of Medical Sciences, Mashhad, Iran. *; f *Department of Epidemiology and Biostatistics, School of Public Health, Tehran University of Medical Sciences, Tehran, Iran.*

**Keywords:** Phytoestrogens, Soy, Isoflavones, vaginal atrophy, meta-analysis

## Abstract

The aim of this study was to perform a meta-analysis of high-quality, randomized, controlled trials (RCTs), to investigate the effectiveness of phytoestrogens in alleviating the menopausal symptoms (vaginal atrophy). Variety of databases including PubMed, Scopus, and Cochrane Central Register of Controlled Trials (CCRCT) were searched up to May 2015 according to the below-mentioned pre-specified search strategy and using the relevant MeSH terms. The mean difference was applied as an estimate of the main effect size. Moreover, due to the considerable heterogeneity among studies, the random-effects model was used to obtain the pooled effect size derived from primary studies. Results showed that while the standardized mean difference of vaginal cell maturation index was increased up to 0.164 percent (with the confidence interval at 95%: (-0.419-0.746), but this increase was not statistically significant (*P*=0.582). The absence of the publication bias was confirmed using the Egger’s regression intercept test (*P* = 0.24). Also, meta-analysis of soybeans studies showed that while the standardized mean difference of vaginal maturation index increased 0.072% in (95% CI: -0.42 to 0.5.), this increase was not statistically significant (p = 0.777). The results confirm that soybeans and phytoestrogens have non-significant positive effects on the vaginal atrophy index. Hence, it is suggested that with regard to non-significant positive effects, non-hormonal treatments along with other treatments such as the vaginal gels and so on should be used more in cases with non-severe vaginal atrophy.

## Introduction

Menopause is described by a decline in estrogen levels, which activates the uncomfortable symptoms of hot flushes, night sweats, sleep disorders, and vaginal atrophy. Among these signs, vaginal dryness and vaginal atrophy are reported by many women to be the most annoying (1). Vaginal atrophy is a chronic and progressive condition characterized by thinning, drying and inflammation of the vaginal walls due to lower level of estrogen (2, 3). About 50 percent of women experience vaginal atrophy, also referred to as genitourinary syndrome of menopause, during and after the menopause; its symptoms include dyspareunia, irritation, bleeding during intercourse, burning and itching (4, 5). Despite other symptoms derived from hypoestrogenemia, like flashing, this condition would not tend to diminish over time (2). This condition wields a significant effect on their attitudes towards sexuality and healthcare and on their quality of life (6, 7). In spite of a wide range of effective hormonal and non-hormonal treatments available to relieve vaginal atrophy symptoms and improve quality of life, many women are unaware of their existence or are unwilling to use them (8). However, it is well understood that hormone replacement therapy (HRT) has some disadvantages and deals with side effects that could be challenging. Lack of compliance and fearing of cancer make patients leave the treatment regimen (9, 10).

In comparison with other menopausal complaints, lower estrogen levels are needed to keep up vaginal health and this could be a positive therapeutic aspect of this condition (11). Both systemic and local estrogen derivatives can alleviate vaginal dryness symptoms and restore vaginal moisture (12). Consequently, introduction of plant derived alternatives for hormone replacement therapy, provide another chance to treat this condition. Phytoestrogens are plant compounds with estrogen-like properties (13). Two major groups of phytoestrogens are isoflavones and lignans; soybeans are affluent in isoflavones, and lignans are found in flaxseed, whole grains, legumes, fruits, and vegetables (14). Soy, red clover, and flaxseed based products are excellent source of these compounds (13, 15-17). 

Although administration of such these is increasing in these days, no consensus has been reached and most of the studies are inconclusive (18). The aim of this study was to perform a meta-analysis of high-quality, randomized, controlled trials (RCTs), to investigate the effectiveness of phytoestrogens in alleviating the menopausal symptoms (vaginal atrophy).

*Methodology*

In the current study, the Preferred Reporting Items for Systematic Reviews and Meta-analysis (PRISMA) guidelines were followed to report a systematic review and meta-analysis (for details on further use, see the PRISMA website, www.prisma-statement.org).

*Information Resources and Search Strategies*

Variety of databases including PubMed, Scopus, and Cochrane Central Register of Controlled Trials (CCRCT) were searched according to the below-mentioned pre-specified search strategy and using the relevant MeSH terms. The databases were searched for articles published up to the end of May 2015, regardless of any limitation such as the language barrier.

*Search Strategy*

In the present study, the search strategy followed was as follows: (Vagina) AND (complementary treatments OR alternative treatments OR nonhormonal OR phytomedicine herbal treatments OR herbs OR red clover OR soy).

***Inclusion Criteria***

Evaluating the effectiveness of non-hormonal or herbal treatments on the alleviation of vaginal atrophy in postmenopausal women and considering the following criteria, all the studies were chosen to be involved in a systematic review. These criteria were as follows:

1. Postmenopausal women participating in the study ought to have at least one year of amenorrhea.

2. At least one of the following indexes is presumed to be reported for the patients with atrophic vaginitis:

a. Vaginal atrophy should be reported by the vaginal cell maturation index.

b. The study should be designed as a randomized, controlled clinical trial.

c. The studied patients should not simultaneously take other hormonal or non-hormonal medications.

d. The study should report the required quantitative data to do a meta-analysis.

*Interventions*

The interventions of this study included prescribing non-hormonal or herbal drugs to alleviate vaginal atrophy in postmenopausal women.

*Implications*

In this meta-analysis study, the vaginal cell maturation index (as a measure of vaginal atrophy) was considered as the primary outcome measurement.

*Data Extraction*

**Figure 1:**
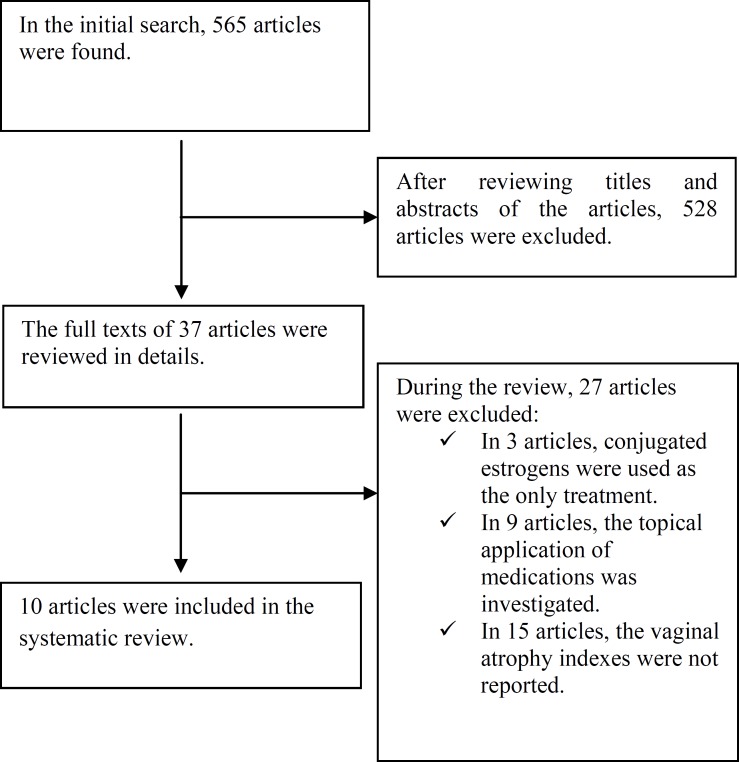
A flow chart depicting the stages of retrieving articles and checking eligibility criteria for meta-analysis

At first, general interest databases consisting of Web of Science, PubMed, Scopus, Google scholar, and Cochrane Library hosting the Central Register of Controlled Trials (CENTRAL) were searched and the search results were entered in the initial list by the researchers. Then, an in-depth research was conducted on the treatments for vaginal atrophy through subject-specific databases. Moreover, in Scopus and PubMed, the key journals related to the issue at hand were manually searched for the content of tables. The researchers made use of the list of references of the chosen articles. In addition, some experts and authors of the articles were consulted in this study. The PRISMA checklist was used to guide the reporting of the systematic review. The search results were entered in Figure 1. Data collection and analysis were done using the *Cochrane Handbook for Systematic Reviews of Interventions*.

In the next phase of the study, two reviewers (M. G. and E. H.) independently screened articles obtained from the search. Initially, in order to attain the eligibility criterion, the titles and abstracts obtained in the search were scrutinized by considering inclusion criteria; thus, the articles which did not meet the inclusion criteria were excluded from the study. All the articles and the tables included in them were carefully scrutinized so as to determine the design of the study and necessary information to evaluate interventions. The reference lists of the articles were reviewed to find out any related article and include it in the study. Any disagreement on the above-mentioned articles between the two reviewers was resolved after additional discussion until a consensus was reached.

*Assessing the Quality of Eligible Studies*

In the research team, two reviewers were selected to assess the quality of the articles by the use of the CONSORT (CONsolidated Standards of Reporting Trials) checklist which was a version of the 2001 CONSORT checklist for reporting trials with medicinal herbs. This checklist was reviewed and changed to some extent. After the two reviewers reached a consensus, the 22-item checklist was utilized to assess the quality.

Data extraction was conducted independently by two reviewers, and any discrepancy discussed until agreement was reached. Besides, at the beginning of the extraction process, after discussing any disagreement, the two reviewers with the assistance of a field supervisor entered into agreements with each other and the designed form was used for data extraction. The extracted data consisted of the year of publication, number of patients in the two segregated groups, age range, duration of menopause, type and dose of herbal treatment, attrition rate, duration of treatment, and the measured values of the desired outcome.

*Statistical Analysis*

The quantitative data extracted from the compiled articles were analyzed and summarized using the Comprehensive Meta-analysis (CMA) software. In this meta-analysis, the vaginal cell maturation index as a measure of vaginal atrophy was considered to be the primary outcome measurement. The sample size, average values before and after the treatment, and standard deviation values of the measured outcome were needed to perform a meta-analysis.

The mean difference was applied as an estimate of the main effect size. Moreover, due to the considerable heterogeneity among studies, the random-effects model was used to obtain the pooled effect size derived from primary studies.

Furthermore, the results of the meta-analysis are graphically displayed in the forest plot. To assess the presence (or absence) of heterogeneity, the Cochran’s Q value was calculated, and *P* <0.05 was considered significant. The I^2^ index was also used to determine the amount of heterogeneity. If there was significant heterogeneity, the sensitivity analysis, meta-regression, and sub-group analysis were used as a standard way to investigate the causes of this heterogeneity.

For each analysis, the publication bias was detected using the funnel plot and Egger’s regression intercept tests.

## Results

After a systematic search of electronic databases, 565 potentially relevant articles were identified. After eliminating duplicate results and reviewing the titles and abstracts, the researchers selected 37 articles. After reviewing the full text of the articles, 27 articles which did not meet the eligibility criteria were excluded from the study. Finally, 10 articles meeting the inclusion criteria were entered into this systematic review. Figure 1 demonstrates the operational scheme of the search procedure of this systematic review.

All the 10 selected articles contained the trials evaluating the effectiveness of soy and other phytoestrogens.

**Table 1:** Characteristic of included studies in meta-analysis.

**Author(s)** **&** **Year of Publication**	**Treatment Duration** **(Week(s))**	**Age** **(Mean years)**	**Attrition Percent**	**Isoflavone ** **Dose (mg)**	**Protein**	**Type of Treatments**	**Type of Control Group**	**No. of patients in treatment groups**	**No. of patients in control groups**	**Randomization Technique**	**Blinding**	**Intention-to-treat reporting**	**Two groups were similar before the treatment **
Baird *et al*., 1995	4	45-65	6%	165	No	Protein	Regular regimen	63	24	No	Yes	Yes	No
D’Anna *et al*., 2007	48	50-70		27	No	A tablet containing isoflavone supplement	placebo	198	191	Yes	Yes	Yes	Yes
Knight *et al*., 2001	12	53	16%	134	Yes	An isoflavone supplementation in the form of a powdered drink	Placebo	9	11	Yes	Yes	Yes	Yes
Radhakrishnan *et al*., 2009	24	53	15%	75	Yes	Soy Powder	Placebo	44	41	Yes	Yes	Yes	Yes
Chiechi *et al*., 2003	24	53	43%	47	Yes	Protein	Regular regimen	22	41	No	Yes	No	Yes
Levis *et al*., 2011	52 to 104	52	43%	200	No	Soy tablet	Placebo	81	71	Yes	Yes	Yes	Yes
Murkies *et al*., 1995	6 to 12	54	18%	Unidentified	No	Soy flour supplementation	Wheat flour	23	24	Yes	Yes	No	Yes
Carmignani *et al*., 2010	16	51	0	90	Yes	Soy Powder	Placebo	20 patients received conjugated estrogen & 20 patients received soy	20	Yes	Yes	Yes	Yes
Knight *et al*., 1999	12	51	5%	Low dose of 40 mgs / High dose of 160 mgs	-	Red clover	Placebo	13 patients in high-dose group & 12 patients in low-dose group	12	Yes	Yes	No	Yes
Colli *et al*., 2012	24	53	17%	100 mgs of flaxseed lignan extract/ flaxseed bread/ 270 mgs of lignans	-	Flaxseed	Placebo	56 patients using flaxseed extract/ 54 patients using flaxseed bread	56	Yes	Yes	No	Yes

*Phytoestrogens*

Ten clinical trials had enough data to enter the meta-analysis (16, 19-27). According to the results of meta-analysis of these studies, the standardized mean difference of vaginal cell maturation index was increased up to 0.164 percent (with the confidence interval at 95%: (-0.419-0.746), but this increase was not statistically significant (*P*=0.582).

**Figure 2:**
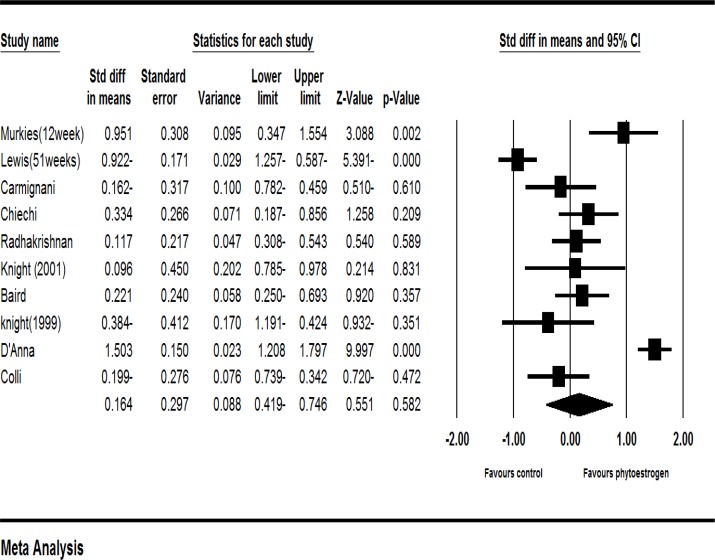
Meta-analysis of studies on the effects of phytoestrogens on the vaginal atrophy index

The results showed strong evidences of heterogeneity (I^2^=93%, *P*=0.582). To investigate the cause of heterogeneity, a sensitivity analysis was performed. In this analysis, excluding each of the studies made no statistically significant changes at the level of significance. Moreover, the studies were classified based on the type of treatment (soy, red clover, flaxseed, and phytoestrogen genistein). The sensitivity analysis based on the type of treatment used in the study indicated that excluding soy reduced the heterogeneity. In other words, with the heterogeneity of 83% and a significance level of *P* <0.001, the study related to soy was identified as the cause of heterogeneity (I^2^=83.1%, P=0.001).

**Figure 3:**
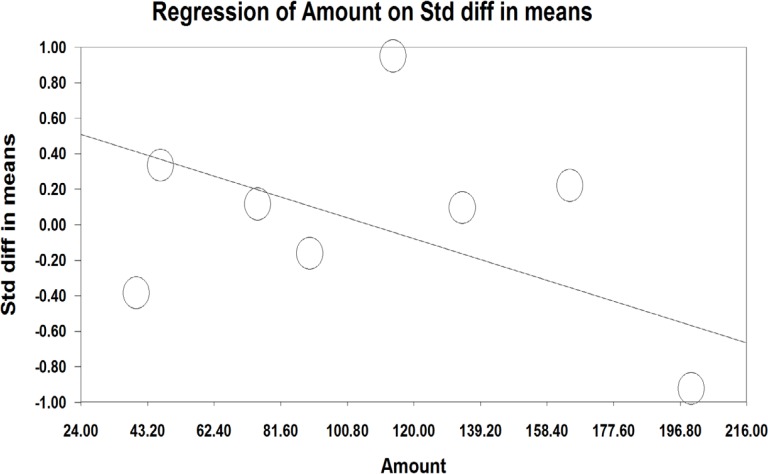
Meta-regression based on the dose of isoflavones in the studies on the effect of phytoestrogens on the vaginal atrophy index

**Figure 4:**
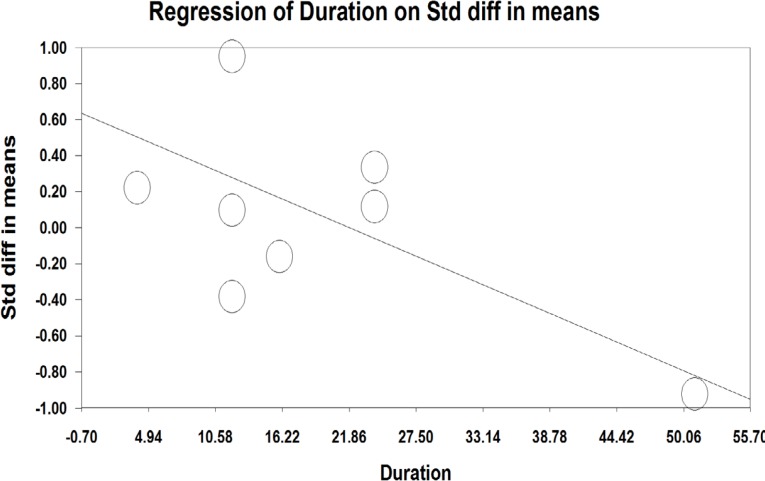
Meta-regression analysis based on the duration of using prescribed isoflavones in studies about the effects of phytoestrogens on the vaginal atrophy index

A meta-regression was also used to assess heterogeneity. The results of meta-regression showed that the treatment duration and isoflavones doses were two important factors causing heterogeneity. According to the results of the meta-regression (Figure 3), as the study was prolonged (-0.029, *P*=0.00001 and -0.0010, *P*<0.001), the effectiveness of isoflavones was significantly decreased (Figure 3 and 4).

**Figure 5:**
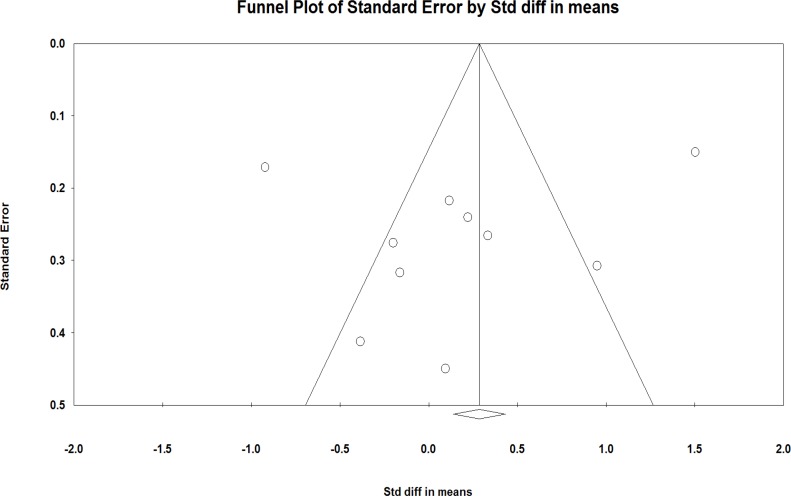
Funnel plot detailing the publication bias in the studies reporting the effect of phytoestrogens on the vaginal atrophy index

No asymmetry was observed in the funnel plot of the effects of phytoestrogens on the vaginal atrophy in the studies (Figure 5).

The absence of the publication bias was confirmed using the Egger’s regression intercept test (*P* = 0.24).

*Soy*

Seven clinical trials had sufficient data for inclusion in the meta-analysis (19-25). Including 494 samples who used soy with dose of 47 to 200 mgs per day for 4 to 53 weeks, the meta-analysis of seven studies revealed that the standardized mean difference of vaginal cell maturation index was increased up to 0.072 percent (95% CI: -0.429-0.573). However, this increase was not statistically significant (*P* =0.77). The findings showed strong evidences of heterogeneity (*P*=0.00; I^2^ = 85.15%)).

**Figure 6:**
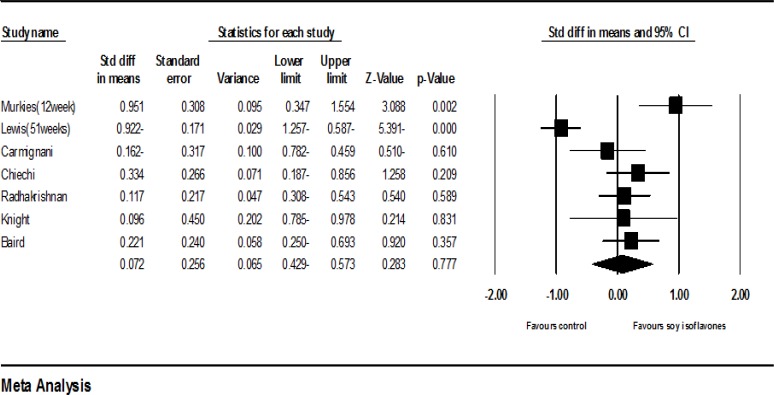
Meta-analysis of the studies on the effects of soy on the vaginal atrophy index

In order to investigate heterogeneity, studies were classified into subgroups based on the study duration (less than or equal to 12 weeks and more than 12 weeks), dose of isoflavones (less than or equal to and greater than 90 mgs), and soy type (soy with or without protein). The analysis of subgroups based on the study duration unexpectedly revealed that studies with a duration of less than or equal to 12 weeks compared with those with a duration of more than 12 weeks could increase the vaginal cell maturation index. Although the comparison between the two doses did not show a significant difference (*P* = 0.34), the subgroup analysis according to the dose of isoflavones also unexpectedly demonstrated that doses less than or equal to 90 mgs compared to doses greater than 90 mgs had managed to increase this index. However, the comparison between the two doses did not show a significant difference (*P* = 0.4). The results based on the soy type showed that the studies in which soy protein was used instead of soy without protein the increase of vaginal cell maturation index was higher, though, the difference between the two groups was not significant (*P* = 0.39).

**Table 2:** Effects of soy on the vaginal atrophy index in terms of the dose of isoflavones, study duration, and type of soy.

**Variable**		**Number of clinical trial studies**	**Number of treatment groups **	**Number of control groups **	**Test of heterogeneity**		**P value**	**Random effect model**
					P	I^2^		Confidence interval of 95%
Soy type	Soy	3	167	119	<0.001	94	0.91	0.006(-1.16-1.03)
	Soy protein	4	95	113	0.69	0	0.39	0.11(-0.38-0.15)
	Comparison test between the two groups							*P*=0.39
Study duration/ week(s)	≤12 weeks(9)	3	118	59	0.12	51	0.09	0.044(-0.97 -0.07)
	>12 weeks	4	167	173	<0.001	86	0.59	-0.17(-0.45- 0.80)
	Comparison test between the two groups							*P*=0.34
Dose of isoflavones	≤90 mgs	3	86	102	0.48	0	0.4	0.12 (-0.041 -0.16)
	≥90 mgs	4	176	130	<0.001	91	0.88	0.06 (-0.96-0.83)
	Comparison test between the groups							*P*=0.4

A meta-regression was also used to assess heterogeneity. The meta-regression showed the study duration and soy dose were two important factors in creating heterogeneity. Based on the meta-regression results, as the study duration (-0.0072, P = 0.00001) and the soy dose (-0.029, P <0.001) were increased, the effectiveness of soy group was significantly reduced.

**Figure 7:**
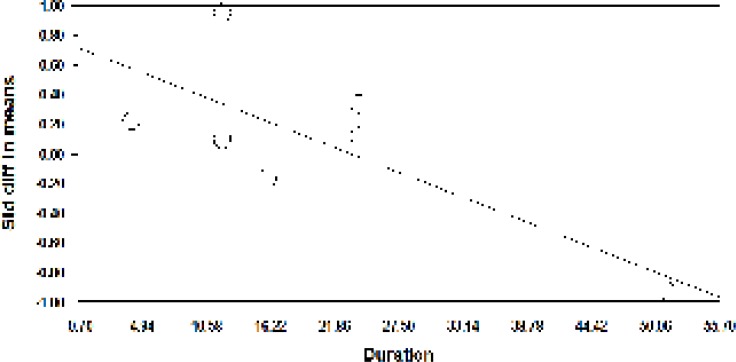
Meta-regression based on the study duration in studies related to the effects of soy on the vaginal atrophy index

**Figure 8:**
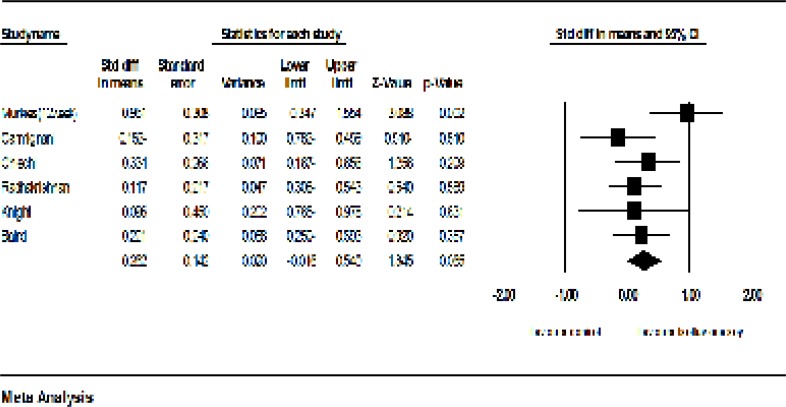
Meta-analysis of studies on the effects of soy on the vaginal atrophy index after the exclusion of Levis *et al*.’s study

The sensitivity analysis showed that the exclusion of Levis *et al*.’s study (20) may cause considerable changes in the level of significance (*P* = 0.065). Furthermore, after the exclusion of Levis *et al*.’s study, the heterogeneity of studies was decreased from 83 percent to 33 percent (*P* = 0.183; I^2^ = 33%, Figure 8). The exclusion of other studies did not make any significant changes in both the effectiveness of soy on the vaginal atrophy and heterogeneity.

There was an asymmetry in the funnel plot of studies on the effects of soy on the vaginal atrophy. The Egger’s test confirmed the presence of publication bias (*P* = 0.06).

**Figure 9:**
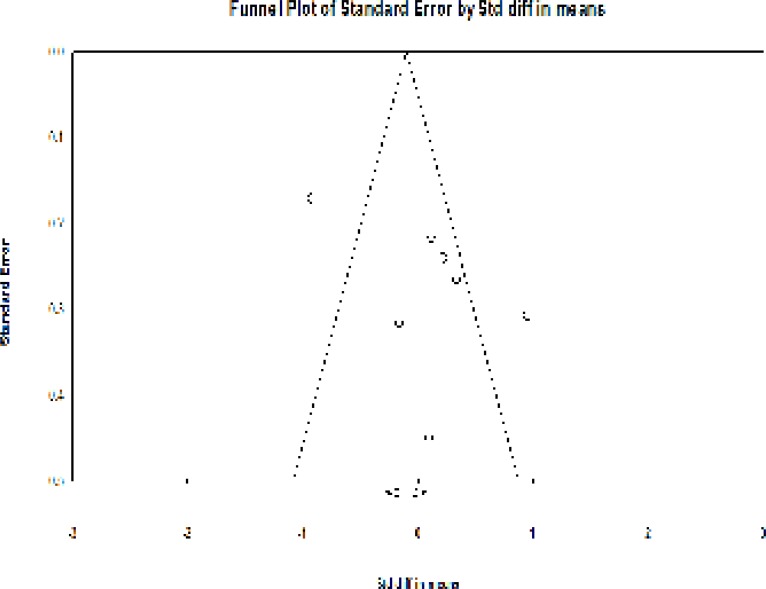
Funnel plot detailing the publication bias in the studies reporting the effect of soy on the vaginal atrophy index

In addition to the seven studies included in the meta-analysis, 3 studies including 176 samples had no sufficient data for inclusion in the meta-analysis (28-30). It should be mentioned that all the authors of the studies were contacted, but unfortunately no responses were received.

## Discussion

The results of the present study show that although medicinal herbs containing phytoestrogens have positive effects on the vaginal atrophy index, these effects have not been reported to be statistically significant. Another meta-analysis results on the menopausal symptoms and side effects of using medicinal herbs indicates phytoestrogens cannot efficiently change the Kupperman index, but they perform better in reducing the flushes of the intervention group than the placebo one (18). Phytoestrogens are sterol molecules produced by plants, and their chemical structure is similar to that of estrogens. One of the main phytoestrogens, which is used in various forms in contemporary societies, is soybean consisting of genistein, daidzein, and glycitein phytoestrogens. In the review study conducted by Hooper and others in 2009, it has been shown that the treatment in which phytoestrogens (especially soy and isoflavones) are used can lead to a non-significant increase in the estradiol level of blood circulation (31). Since higher estradiol levels of blood circulation can also upsurge the risk of breast, endometrial, and ovarian cancers, so more studies should be conducted to explore the side effects of such medications. Furthermore, according to studies on soy phytoestrogens, prescribing this phytoestrogen cannot make any changes in endometrial thickness and its effect on the endometrium is mostly antagonistic. Likewise, according to previous studies, flaxseeds (lignans) enjoy a protective effect against breast cancer (32). Numerous studies have been done on the advantages and disadvantages of soybeans, for example in one of the studies it is suggested that consuming soybeans may reduce the risk of cardiovascular diseases through different mechanisms. Therefore, by reducing the harmful cholesterol levels in the blood and being rich in omega 6 and 3 as well as soflavones, soybeans enhance the endothelial function and ultimately reduce the risk of atherosclerosis. Still, sufficient grounds on the reduction of the risk of breast cancer by soybeans have not been presented in different studies (33-35). In another review study conducted in 2016 which reviews the studies published up to 2013, it is approved that soy isoflavones may improve symptoms and signs of the vaginal atrophy in postmenopausal women with the condition (36). However, in different studies, the beneficial effects of this substance are still under questioned due to the heterogeneity of compiled studies; thus, the need for more evidence is irrefutable. In the current study, both the quantitative results (the meta-analysis) and the qualitative results with positive (non-significant) effects are presented in the soy group compared with the control group. In this study, the removal of the study of Levis and others, to a large extent, lead to a decrease in the heterogeneity of the studies on soybean. Besides, at the same time the effect size is also increased. It is demonstrated that the dose of soy isoflavones and the duration of the intervention may have implications to reduce the vaginal atrophy index happened thanks to the effects of the dominant antiestrogen with doses higher than 90 mgs and for more than 12 weeks. A lot of studies have been conducted on investigating the effects of phytoestrogens on improving symptoms of menopausal period. Thus, through a PubMed search with the keywords of “menopause AND phytoestrogen”, numerous studies can be found. However, the main problem is the heterogeneity and very different results reported in the studies (37, 38).

Lignans are one of the main classes of phytoestrogens which are not investigated in these articles. The results of studies already reported have not shown any evidence in favor of this class of phytoestrogens compared to the placebo group. For instance, in the study conducted by Simbalista and others, regarding the reduction in menopausal symptoms, it is reported there are no significant differences between those who daily receive 46 mgs Lignan and those who have their daily routine (less than 1 mg) and both groups experience a reduction in symptoms (39).

Similar to other studies, this study also has its own limitations which should be noted. One of the limitations of the studies on phytoestrogens is the inadequate assessment of patients’ compliance with medications or placebos. So, in some studies, assessing compliance has just been based on counting pills or medication and placebo packages, but urinary isoflavone levels are not measured in these studies. Accordingly, the possibility of unintentional or intentional use of isoflavones in the regimen of control group is not measurable. To address this problem, it is suggested that future studies attempt to assess compliance through not only counting packages and the remaining pills but also investigating urinary isoflavone levels at baseline, during, and after the treatment. If there are significant changes in the control group before and after the treatment, the presence of publication bias of the study should be reported. Another limitation of the studies on phytoestrogens is high heterogeneity which may be occurred due to different types or different effects of phytoestrogens, different dosages of isoflavones in various products, and the type of control group.

In this systematic review, the effects of non-hormonal treatments on the vaginal atrophy index are investigated. The results confirm that soybeans and phytoestrogens have non-significant positive effects on the vaginal atrophy index. Hence, it is suggested that, with regard to non-significant positive effects, non-hormonal treatments along with other treatments such as the vaginal gels and so on should be used more in cases with non-severe vaginal atrophy. However, given the fact that in some studies it has been found out phytoestrogens can increase the level of sex hormones (much lower than the levels induced by the hormone therapy), so precautions should be taken to use them in individuals with contraindications. Thus, further studies are needed in this field.
